# Hepatocellular carcinoma is the most common liver-related complication in patients with histopathologically-confirmed NAFLD in Japan

**DOI:** 10.1186/s12876-018-0900-1

**Published:** 2018-11-06

**Authors:** Norio Akuta, Yusuke Kawamura, Yasuji Arase, Satoshi Saitoh, Shunichiro Fujiyama, Hitomi Sezaki, Tetsuya Hosaka, Masahiro Kobayashi, Mariko Kobayashi, Yoshiyuki Suzuki, Fumitaka Suzuki, Kenji Ikeda, Hiromitsu Kumada

**Affiliations:** 1 Department of Hepatology, Toranomon Hospital and Okinaka Memorial Institute for Medical Research, 2-2-2 Toranomon, Minato-ku, Tokyo, 105-0001 Japan; 2 Liver Research Laboratory, Toranomon Hospital, Tokyo, Japan

**Keywords:** Nonalcoholic fatty liver disease, Nonalcoholic steatohepatitis, Hepatocellular carcinoma, Liver-related events, Cardiovascular events, Type 2 diabetes mellitus, Malignancy, Mortality, Fibrosis stage, Hepatocyte steatosis

## Abstract

**Background:**

The incidence of liver-related events, cardiovascular events and type 2 diabetes mellitus in patients with histopathologically confirmed NAFLD remains unclear.

**Methods:**

We retrospectively investigated the incidence of liver events, cardiovascular events, malignancy, and type 2 diabetes mellitus in 402 Japanese patients with histopathologically confirmed NAFLD for a median follow-up of 4.2 years. We also investigated predictors of the development of hepatocellular carcinoma and type 2 diabetes mellitus in these patients.

**Results:**

The rate of liver-related events per 1000 person years was 4.17 (hepatocellular carcinoma, 3.67; hepatic encephalopathy, 1.60; esophago-gastric varices, 2.43; ascites, 0.80; and jaundice, 0.40). The rate of cardiovascular events and type 2 diabetes mellitus was 5.73 and 9.95, respectively. Overall mortality was 3.33 (liver-related events, 1.25; cardiovascular events, 0.42; and malignancies other than hepatocellular carcinoma, 0.83), in patients free of previous or current malignancies. Multivariate analyses identified old age (≥70 years) and advanced fibrosis stage 4 as significant determinants of hepatocellular carcinoma development, and hepatocyte steatosis (> 33%), female sex, and serum ferritin (≤80 μg/l) as significant determinants of type 2 diabetes mellitus development in these patients.

**Conclusions:**

Our results highlighted the importance of cardiovascular and liver-related events in Japanese patients with histopathologically-confirmed NAFLD. Hepatocellular carcinoma was the most common liver-related event, and the incidence of hepatocellular carcinoma was more than half of that of cardiovascular events.

## Background

The most common liver disease worldwide is non-alcoholic fatty liver disease (NAFLD) [1–6]. Liver pathology ranges from the typically benign non-alcoholic fatty liver to non-alcoholic steatohepatitis (NASH), which may progress to liver cirrhosis, hepatocellular carcinoma (HCC), and liver failure [7].

The incidence of liver events, cardiovascular events, malignancy, and type 2 diabetes mellitus (T2DM) in patients with histopathologically confirmed NAFLD remains unclear. T2DM and fibrosis stage are significant and independent risk factors for HCC in patients with NAFLD [5]. Results of recent prospective studies have shown that antidiabetic drugs may improve histological features, including fibrosis stage [8–10]. Thus, it may be important to identify predictors of the development of HCC and T2DM to improve the prognosis of patients with NAFLD.

It has been suggested that fibrosis stage may be more reliable than the NAFLD activity score (NAS) for the prediction of liver-specific mortality [11]. Fibrosis stage, but not other histopathological features of steatohepatitis, was reported to be an independent and significant predictor of overall mortality, liver transplantation, and liver-related events [12].

The purpose of the present study was to determine the incidence of liver-related events, cardiovascular events, and T2DM, and the predictors of development of HCC and T2DM in patients with NAFLD by retrospectively analyzing the outcome of 402 Japanese patients with histopathologically confirmed NAFLD.

## Methods

### Patients

This is a retrospective cohort study of patients with histopathologically-confirmed NAFLD. Between 1976 and 2017, liver biopsy was performed at our hospital for patients with liver dysfunction and/or fatty liver diagnosed by abdominal ultrasonography. Of those, the diagnosis of NAFLD was confirmed in 402 patients by histopathology. The median duration of follow-up, from diagnosis to death or last visit, was 4.2 years (range, 0.0–41.4 years), and the total sum of person-years was 2625 years. The characteristics of the patients at the time of histopathological diagnosis of NAFLD are summarized in Table 1. Patients with histopathological changes of steatosis in at least 5% of hepatocytes and alcohol intake < 20 g/day were included in the analysis. We excluded patients with 1) underlying liver disease (e.g., viral hepatitis, autoimmune hepatitis, drug-induced liver disease, or primary biliary cirrhosis); 2) systemic autoimmune diseases (e.g., systemic lupus erythematosus and rheumatoid arthritis); and 3) metabolic diseases (e.g., hemochromatosis, α-1-antitrypsin deficiency, or Wilson disease).

**Table 1:** Patient characteristics at the time of histological diagnosis of NAFLD

Demographic data	
Numbers of patients	402
Gender, Male / Female, n	245 / 157
Age, y^a^	51 (20–87)
Body mass index, kg/m^2a^	26.1 (18.1–42.4)
Presence of previous and current malignancy	
None / Hepatocellular carcinoma / Other malignancy, n	351 / 26 / 30
Type 2 diabetes mellitus, Absence / Presence, n	276 / 126
Hypertension, Absence / Presence, n	230 / 172
Hyperlipidemia, Absence / Presence, n	274 / 128
Histological findings	
Steatosis, 5–33% / > 33–66% / > 66%, n	152 / 149 / 98
Lobular inflammation	
No foci / < 2 foci / 2–4 foci / > 4 foci per 200× field, n	28 / 242 / 116 / 13
Ballooning, None / Few cells / Many cells, n	39 / 252 / 108
Stage, 0 / 1 / 2 / 3 / 4, n	48 / 165 / 63 / 98 / 28
NAFLD activity score, ≤2 / 3, 4 / ≥5, n	34 / 181 / 184
Diagnosis according to FLIP algorithm, NASH / non-NASH, n	349 / 50
Laboratory data^a^	
Serum aspartate aminotransferase, IU/l	44 (3–378)
Serum alanine aminotransferase, IU/l	69 (15–783)
Gamma-glutamyl transpeptidase, IU/l	72 (11–990)
Platelet count, ×10^3^/mm^3^	213 (40–471)
Fasting plasma glucose, mg/dl	101 (65–287)
HbA1c, %	5.9 (4.4–12.6)
Uric acid, mg/dl	5.9 (1.9–11.1)
Total cholesterol, mg/dl	204 (101–370)
Triglycerides, mg/dl	140 (31–1088)
High-density lipoprotein cholesterol, mg/dl	45 (14–85)
Low-density lipoprotein cholesterol, mg/dl	120 (27–243)
Serum ferritin, μg/l	227 (< 10–2067)
High sensitive C-reactive protein, mg/dl	0.095 (0.006–2.240)
Alpha-fetoprotein, μg/l	4 (1–10,930)
PIVKA-II, AU/l	18 (1–157,050)

Data are number of patients, except those denoted by ^a^, which represent the median (range) values

The study was conducted in compliance with the International Conference on Harmonisation guidelines for Good Clinical Practice (E6) and the 2013 Declaration of Helsinki. The protocol was approved by the institutional review board at Toranomon Hospital (number 953). Written informed consent was provided by all patients prior to liver biopsy.

### Diagnosis and follow-up

Liver-related events included HCC, hepatic encephalopathy, esophago-gastric varices with bleeding, ascites, and jaundice. Cardiovascular events included coronary artery disease, heart valve disease, arrhythmia, heart failure, hypertension, orthostatic hypotension, shock, endocarditis, diseases of the aorta and its branches, disorders of the peripheral vascular system, and stroke. Furthermore, the incidence of T2DM and other malignancies, apart from HCCs, were also evaluated. The incidence of T2DM was assessed at least twice a year after baseline examination. T2DM was diagnosed as the presence of elevated fasting plasma glucose (≥126 mg/dl), elevated HbA1c (≥6.5%) or self-reported history of clinical diagnosis.

Hematological and biochemical data were collected at least twice yearly after the diagnosis of NAFLD. Ultrasonography, computed tomography, or magnetic resonance imaging studies were performed at least once annually.

**Fig. 1:**
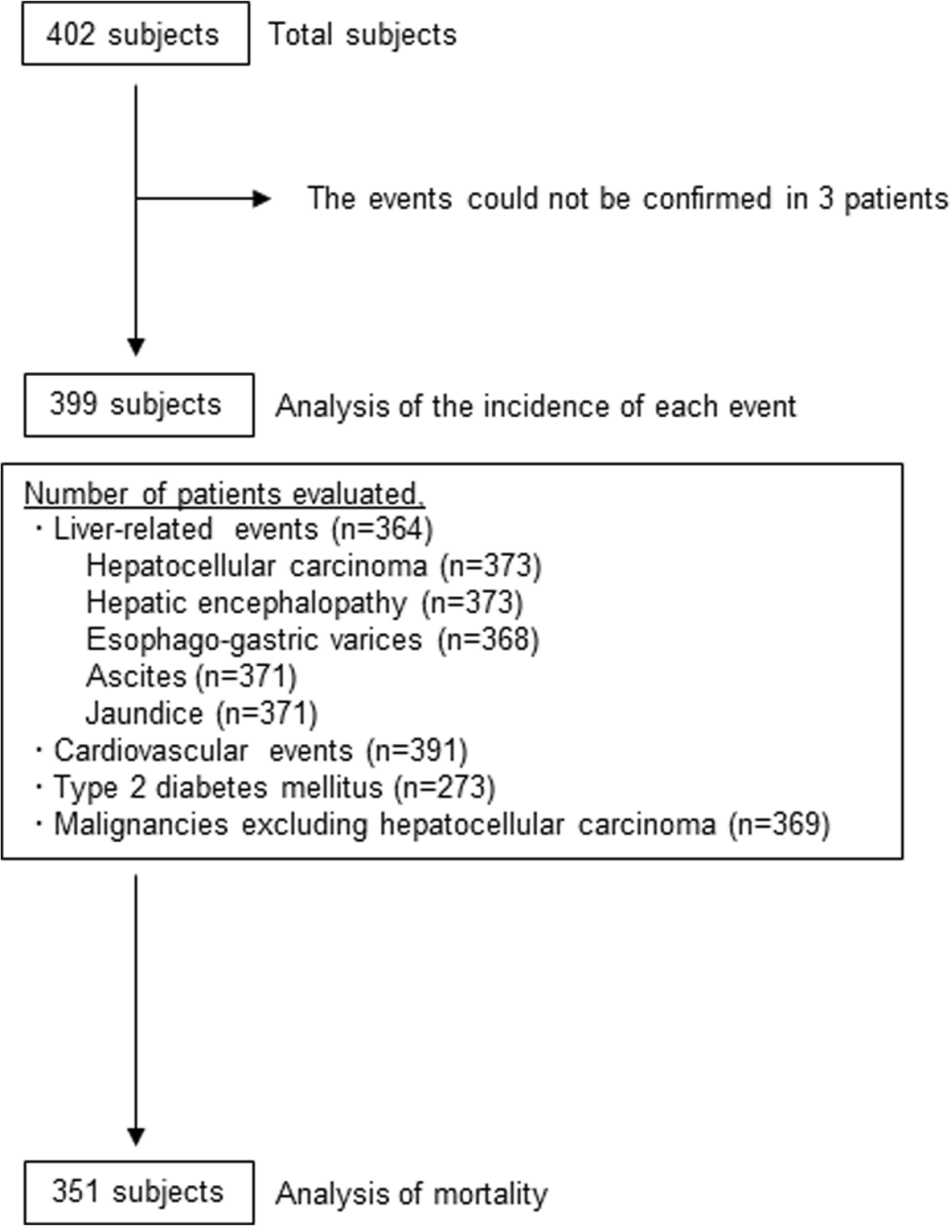
Between 1976 and 2017, liver biopsy was performed at our hospital, for patients with liver dysfunction and/or fatty liver, as confirmed by abdominal ultrasonography. NAFLD was confirmed histopathologically in 402 Japanese consecutive patients. Follow-up was missing in 3 patients, and 399 patients were evaluated in sub-cohorts in which patients who had experienced an event were excluded from that specific analysis. Mortality was evaluated in 351 patients, without previous or current malignancies at the time of NAFLD diagnosis

The clinical details of the events of 3 patients were missing. The rate of cancer development was evaluated only in patients confirmed to have no previous or existing HCC at the time of diagnosis of NAFLD, and no previous or current other malignancies apart from HCCs. The rates of development of hepatic encephalopathy, esophago-gastric varices, ascites, and jaundice were evaluated in patients confirmed to have no previous or current hepatic encephalopathy, esophago-gastric varices, ascites, jaundice or HCC at the time of NAFLD diagnosis, respectively. Mortality was evaluated in patients, who had no previous or present malignancies at the time of NAFLD diagnosis. Details of patient enrolment are shown in Fig. 1.

### Liver histopathology

Liver specimens were obtained with a 14-gauge modified Vim Silverman needle (Tohoku University style, Kakinuma Factory, Tokyo, Japan), a 16-gauge core tissue biopsy needle (Bard Peripheral Vascular Inc., Tempe, AZ) or surgical resection. Specimen was fixed in 10% formalin, and the prepared sections were stained with hematoxylin-eosin, Masson trichrome, silver impregnation, or periodic acid-Schiff after diastase digestion. Four pathologists (K.K., F.K., T.F., and T.F.), who were blinded to the clinical findings, evaluated each specimen, and the final assessment was reported by consensus. An adequate liver biopsy sample was defined as a specimen longer than 1.5 cm and/or containing more than 11 portal tracts.

Steatosis grade 0, 1, 2, and 3 corresponded to steatosis of < 5%, ≥5 to < 33%, ≥33 to < 66%, and ≥ 66% of hepatocytes, respectively. Lobular inflammation with no foci, < 2 foci, 2–4 foci, and ≥ 4 foci per 200× field was scored as 0, 1, 2, and 3, respectively. Hepatocyte ballooning of none, few, and many cells was scored as 0, 1, and 2, respectively. The sum of the steatosis, lobular inflammation, and hepatocyte ballooning scores (range, 0–8 points) was the NAS [13]. Fibrosis stage was defined as 0, 1, 2, 3, and 4 [13, 14]. NASH was defined according to the Fatty Liver Inhibition of Progression (FLIP) algorithm [15].

### Clinical parameters

We analyzed clinicopathological parameters that could affect NAFLD prognosis. At our hospital, the normal range of aspartate aminotransferase (AST) was 13–33 IU/l, and the normal range of alanine aminotransferase (ALT) was 8–42 IU/l for males and 6–27 IU/l for females. Obesity was defined as body mass index of > 25.0 kg/m^2^.

### Statistical analysis

The incidence of each event was analyzed during the period from the time of histopathological diagnosis of NAFLD until the last visit or occurrence of event. Stepwise Cox regression analysis was used to determine independent predictive factors associated with the development of HCC and T2DM. The hazard ratio (HR) and 95% confidence interval (95% CI) were also calculated. Variables that were statistically significant on univariate analysis were tested by multivariate analysis to identify significant independent factors. Significance was set at *p* value < 0.05 by the two-tailed test. Statistical comparisons were performed with the SPSS software (SPSS Inc., Chicago, IL, USA).

## Results

### Incidence of liver-related events in NAFLD

During the follow-up, 9/373 (2.4%) patients developed HCC (rate per 1000 person years, 3.67), and 21/369 (5.7%) patients developed malignancies other than HCC (rate per 1000 person years, 8.93).

4/373 (1.1%) patients developed hepatic encephalopathy (rate per 1000 person years, 1.60). 6/368 (1.6%) patients developed esophago-gastric varices (rate per 1000 person years, 2.43). 2/371 (0.5%) patients developed ascites (rate per 1000 person years, 0.80). 1/371 (0.3%) patient developed jaundice (rate per 1000 person years, 0.40).

Hence, 10 of 364 patients (2.8%) confirmed to have no previous or current liver-related events at NAFLD diagnosis developed liver-related events (rate per 1000 person years, 4.17) (Table 2).

**Table 2:** Incidence of liver events, cardiovascular events and type 2 diabetes mellitus in patients with NAFLD

Events	n/N (%)^a^	1000 person years
Liver-related events^b^	10/364 (2.8%)	4.17
Hepatocellular carcinoma	9/373 (2.4%)	3.67
Hepatic encephalopathy	4/373 (1.1%)	1.60
Esophago-gastric varices	6/368 (1.6%)	2.43
Ascites	2/371 (0.5%)	0.80
Jaundice	1/371 (0.3%)	0.40
Cardiovascular events	14/391 (3.6%)	5.73
Type 2 diabetes mellitus	19/273 (7.0%)	9.95
Malignancies except for hepatocellular carcinoma	21/369 (5.7%)	8.93

^a^n; number of events. N; number of patients, not having, or having had, the respective event simultaneously or previously to the time of NAFLD diagnosis

^b^Liver-related events were evaluated in patients, without previous or current hepatocellular carcinoma at the time of NAFLD diagnosis

### Predictors of development of HCC in patients with NAFLD

The characteristics of the 373 patients confirmed to have no previous or current HCC at the time of NAFLD diagnosis were evaluated for prediction of HCC development. Twenty-seven potential predictive factors of the clinicopathological parameters were analyzed (Table 3). Univariate analysis identified the following five parameters that correlated significantly with HCC development: age, fibrosis stage, platelet count, total cholesterol, and α-fetoprotein. These factors were entered into multivariate analysis, which identified two factors that significantly and independently influenced HCC development: advanced age (≥70 years; HR 9.54, 95% CI = 1.63–55.9, *P* = 0.012) and advanced fibrosis stage (stage 4; HR 7.14, 95% CI = 1.29–39.5, *P* = 0.024) (Table 3).

**Table 3:** Predictors of development of hepatocellular carcinoma in patients with NAFLD

Factor	Category	Univariate	(95% CI)	*P* value*	Multivariate	(95% CI)	*P* value*
		Hazard ratio			Hazard ratio		
Demographic data							
Gender	Male	1					
	Female	0.24	(0.03–1.97)	0.186			
Age	< 70 y	1			1		
	≥70 y	18.6	(3.74–92.6)	< 0.001	9.54	(1.63–55.9)	0.012
Body mass index	< 25.0 kg/m^2^	1					
	≥25.0 kg/m^2^	0.46	(0.11–1.88)	0.276			
Type 2 diabetes mellitus	Absence	1					
	Presence	3.64	(0.95–14.0)	0.060			
Hypertension	Absence	1					
	Presence	1.06	(0.28–3.95)	0.932			
Hyperlipidemia	Absence	1					
	Presence	0.36	(0.05–2.91)	0.339			
Histological findings							
Steatosis	5–33%	1					
	> 33%	0.40	(0.10–1.63)	0.201			
Lobular inflammation	< 2 foci per 200× field	1					
	≥2 foci per 200× field	0.31	(0.04–2.50)	0.272			
Ballooning	None / Few cells	1					
	Many cells	0.47	(0.05–3.84)	0.482			
Stage	0–3	1			1		
	4	33.9	(7.14–161)	< 0.001	7.14	(1.29–39.5)	0.024
NAFLD activity score	< 5	1					
	≥5	0.19	(0.02–1.50)	0.114			
Diagnosis according to FLIP algorithm	non-NASH	1					
	NASH	1.34	(0.17–10.8)	0.784			
Laboratory data							
Serum aspartate aminotransferase	< 2 × ULN IU/l	1					
	≥2 × ULN IU/l	2.74	(0.73–10.2)	0.134			
Serum alanine aminotransferase	< 2 × ULN IU/l	1					
	≥2 × ULN IU/l	0.85	(0.22–3.22)	0.810			
Gamma-glutamyl transpeptidase	< 110 IU/l	1					
	≥110 IU/l	1.29	(0.34–4.86)	0.706			
Platelet count	< 200 × 10^3^/mm^3^	1			1		
	≥200 × 10^3^/mm^3^	0.06	(0.01–0.50)	0.009	0.14	(0.01–1.33)	0.086
Fasting plasma glucose	< 110 mg/dl	1					
	≥110 mg/dl	3.61	(0.85–15.3)	0.081			
HbA1c	< 5.8%	1					
	≥5.8%	56.2	(0.01–634,349)	0.397			
Uric acid	< 7.1 mg/dl	1					
	≥7.1 mg/dl	0.03	(0.00–21.0)	0.298			
Total cholesterol	< 200 mg/dl	1					
	≥200 mg/dl	0.18	(0.04–0.87)	0.033			
Triglycerides	< 150 mg/dl	1					
	≥150 mg/dl	0.46	(0.09–2.28)	0.342			
High-density lipoprotein cholesterol	< 41 mg/dl	1					
	≥41 mg/dl	0.58	(0.15–2.25)	0.428			
Low-density lipoprotein cholesterol	< 136 mg/dl	1					
	≥136 mg/dl	0.24	(0.03–2.06)	0.191			
Serum ferritin	< 81 μg/l	1					
	≥81 μg/l	0.71	(0.14–3.61)	0.674			
High sensitive C-reactive protein	< 0.2 mg/dl	1					
	≥0.2 mg/dl	1.02	(0.11–10.0)	0.984			
Alpha-fetoprotein	< 5 μg/l	1			1		
	≥5 μg/l	7.15	(1.44–35.6)	0.016	4.44	(0.84–23.4)	0.079
PIVKA-II	< 21 AU/l	1					
	≥21 AU/l	0.47	(0.06–4.05)	0.495			

*Significance was determined using a Cox proportional hazard model. *CI* confidence interval, *ULN* upper limit of normal

### Rate of development of cardiovascular events in NAFLD

The characteristics of the 391 patients confirmed to have no previous or current cardiovascular events at NAFLD diagnosis were evaluated for the rate of development of cardiovascular events. During the follow-up, 14 patients (3.6%) developed cardiovascular events, and the development rate per 1000 person years was 5.73 (Table 2).

### Rate and predictors of development of T2DM in NAFLD

The incidence of type 2 diabetes mellitus was evaluated in patients confirmed to have no previous or current T2DM (*n* = 273) at the time of NAFLD diagnosis. During the follow-up, 19 (7.0%) patients developed T2DM (rate per 1000 person years, 9.95) (Table 2).

The characteristics of the 273 patients confirmed to have no previous or current T2DM at the time of histopathological diagnosis of NAFLD were evaluated for prediction of T2DM development. Twenty-six potential predictive factors of the clinicopathological parameters were analyzed (Table 4). Univariate analysis identified the following five parameters that correlated significantly with T2DM development: gender, hepatocyte steatosis, γ-glutamyl transpeptidase, low-density lipoprotein cholesterol, and serum ferritin. These factors were entered into multivariate analysis, which identified three factors that significantly and independently influenced T2DM development: gender (female; HR 5.83, 95% CI = 1.47–23.1, *P* = 0.012), hepatocyte steatosis (> 33%; HR 9.52, 95% CI = 1.57–57.6, *P* = 0.014), and serum ferritin (≥81 μg/l; HR 0.18, 95% CI = 0.06–0.56, *P* = 0.003) (Table 4).

**Table 4:** Predictors of development of type 2 diabetes mellitus in patients with NAFLD

Factor	Category	Univariate	(95% CI)	*P* value*	Multivariate	(95% CI)	*P* value*
		Hazard ratio			Hazard ratio		
Demographic data							
Gender	Male	1			1		
	Female	5.59	(2.07–15.1)	0.001	5.83	(1.47–23.1)	0.012
Age	< 70 y	1					
	≥70 y	0.05	(0.00–5618)	0.606			
Body mass index	< 25.0 kg/m^2^	1					
	≥25.0 kg/m^2^	1.41	(0.56–3.57)	0.472			
Hypertension	Absence	1					
	Presence	1.53	(0.62–3.77)	0.357			
Hyperlipidemia	Absence	1					
	Presence	1.20	(0.43–3.56)	0.732			
Histological findings							
Steatosis	5–33%	1			1		
	> 33%	3.30	(1.09–10.0)	0.035	9.52	(1.57–57.6)	0.014
Lobular inflammation	< 2 foci per 200× field	1					
	≥2 foci per 200× field	1.70	(0.63–4.57)	0.296			
Ballooning	None / Few cells	1					
	Many cells	0.57	(0.13–2.49)	0.452			
Stage	0–3	1					
	4	1.47	(0.20–11.0)	0.711			
NAFLD activity score	< 5	1					
	≥5	1.30	(0.51–3.26)	0.583			
Diagnosis according to FLIP algorithm	non-NASH	1					
	NASH	1.42	(0.41–4.90)	0.579			
Laboratory data							
Serum aspartate aminotransferase	< 2 × ULN IU/l	1					
	≥2 × ULN IU/l	1.19	(0.45–3.13)	0.730			
Serum alanine aminotransferase	< 2 × ULN IU/l	1					
	≥2 × ULN IU/l	1.47	(0.59–3.64)	0.408			
Gamma-glutamyl transpeptidase	< 110 IU/l	1					
	≥110 IU/l	0.28	(0.08–0.96)	0.043			
Platelet count	< 200 × 10^3^/mm^3^	1					
	≥200 × 10^3^/mm^3^	1.94	(0.64–5.87)	0.239			
Fasting plasma glucose	< 110 mg/dl	1					
	≥110 mg/dl	1.31	(0.37–4.62)	0.672			
HbA1c	< 5.8%	1					
	≥5.8%	2.33	(0.32–16.9)	0.404			
Uric acid	< 7.1 mg/dl	1					
	≥7.1 mg/dl	1.28	(0.49–3.38)	0.615			
Total cholesterol	< 200 mg/dl	1					
	≥200 mg/dl	2.43	(0.80–7.34)	0.116			
Triglycerides	< 150 mg/dl	1					
	≥150 mg/dl	0.78	(0.31–1.97)	0.594			
High-density lipoprotein cholesterol	< 41 mg/dl	1					
	≥41 mg/dl	0.49	(0.20–1.20)	0.117			
Low-density lipoprotein cholesterol	< 136 mg/dl	1					
	≥136 mg/dl	3.20	(1.06–9.69)	0.040			
Serum ferritin	< 81 μg/l	1			1		
	≥81 μg/l	0.26	(0.10–0.70)	0.008	0.18	(0.06–0.56)	0.003
High sensitive C-reactive protein	< 0.2 mg/dl	1					
	≥0.2 mg/dl	1.84	(0.57–5.97)	0.312			
Alpha-fetoprotein	< 5 μg/l	1					
	≥5 μg/l	1.08	(0.38–3.04)	0.887			
PIVKA-II	< 21 AU/l	1					
	≥21 AU/l	0.23	(0.03–1.79)	0.160			

*Significance was determined using a Cox proportional hazard model. *CI* confidence interval, *ULN* upper limit of normal

### Mortality in NAFLD, without previous or current malignancies

In patients without previous or present malignancies at the time of NAFLD diagnosis, the overall mortality per 1000 person years was 3.33. The rate was 1.25 for those who died of liver-related events, 0.42 for those who died of cardiovascular events, and 0.83 for those who died of malignancies events other than HCC (Table 5). In the 3 patients who died from liver-related events, 2 of 3 patients and 1 of 3 patients had HCC and liver failure, respectively.

**Table 5:** Mortality in patients with NAFLD, without previous and current malignancies

Cause of death	n/N (%)^a^	1000 person years
Overall	8/351 (2.3%)	3.33
Liver-related events	3/351 (0.9%)	1.25
Cardiovascular events	1/351 (0.3%)	0.42
Malignancies events except for hepatocellular carcinoma	2/351 (0.6%)	0.83
Other events	2/351 (0.6%)	0.83

^a^n; number of events. N; number of patients, not having, or having had, the respective event simultaneously or previously to the time of NAFLD diagnosis

## Discussion

The incidence of liver-related and cardiovascular events in patients with histopathologically confirmed NAFLD remains unclear. Furthermore, it is important to identify the predictors of development of HCC and T2DM to improve the prognosis of patients with NAFLD. There is limited information on the long-term development rate of these events in patients with histopathologically confirmed NAFLD [16, 17].

We found that patients with NAFLD were at increased risk of HCC (HR 6.55, *P* = 0.001) and cardiovascular diseases (HR 1.55, *P* = 0.01) [18]. In the present study, cardiovascular events had the highest incidence (5.73 per 1000 person years), with liver-related events the second highest incidence (4.17 per 1000 person years). Interestingly, among liver-related events, HCC was the event with the highest incidence (3.67 per 1000 person years). The incidence of HCC was more than half of that of cardiovascular events (3.67 vs. 5.73 per 1000 person years). In the present study, the mortality of liver-related events per 1000 person years (1.25) was not lower than that of cardiovascular events (0.42) and malignancies other than HCC (0.83). Hence, liver-related events accounted for about one-third of mortality in NAFLD patients who presented with no previous or present malignancies at the time of NAFLD diagnosis.

The present study has certain limitations. First, only a small number of deaths (8 patients) were recorded during the study period. Further studies of larger number of patients with NAFLD and longer follow-up period should be performed to investigate the impact of each event on mortality.

In another study, the incidence of HCC among all malignancies reported in 1600 patients with NAFLD diagnosed based on the presence of fatty liver by ultrasonography, was 6.0%, and the rate per 1000 person years was 0.78, [19]. However, the results of our study indicated that the rate per 1000 person years was 3.67, and rate of HCC was higher compared to the above studies. The discrepant results could reflect patient selection bias, as all patients had histopathologically confirmed NAFLD, with elevated aminotransferases (indicators of high activity) and/or low levels of platelet counts (indicator of advanced fibrosis stage). Furthermore, patients treated with anti-platelet agents and anticoagulants for the prevention of cardiovascular events, did not undergo liver biopsy, and were thus not included. Also, patients who visit the hospital regularly tend to receive treatments for hypertension, hyperlipidemia, and diabetes mellitus, which are as risk factors of cardiovascular events. As previously reported [5], multivariate analysis identified advanced fibrosis stage and old age as significant and independent determinants of HCC development.

Seko and colleagues [20] reported that 13 of 89 (14.6%) patients with biopsy-confirmed NAFLD developed T2DM, and multivariate analysis identified the presence of insulin resistance as an independent risk factor for the development of T2DM. The present study showed that 19 of 273 (6.96%) patients developed T2DM, which is a lower rate compared to the above study. The discrepant results could be due to differences in the diagnostic methods for T2DM; patients in the previous study were diagnosed with a 75-g oral glucose tolerance test. The other reasons for the low frequency of T2DM development is probably that many patients had diabetes at the time of liver biopsy and the follow-up time was short, i.e. selection bias and short follow-up. Interestingly, multivariate analysis in the present study identified higher frequencies of hepatocyte steatosis, lower levels of serum ferritin, and female sex as significant and independent determinants of the incidence of T2DM. Previous reports showed that the incidence of T2DM is higher in postmenopausal female patients with hepatocyte steatosis [17, 21]. However, at this stage, we do not known why lower levels of serum ferritin influence the incidence of T2DM. This finding must be further explored and validated in a larger independent cohort.

Other limitations of the present study included the retrospective study design and the fact that the patients in our study were inpatients. We could not investigate whether factors during the course of observation, such as weight loss and exercise, might affect the development of HCC and T2DM. Furthermore, all participants were Japanese, and thus the results might not be applicable to patients of other races or ethnic groups. Also, the study did not address the epidemiological burden and complexity of the natural history of NAFLD [22, 23]. Identification of predictors of development of HCC and T2DM in patients with NAFLD is a clinical priority due to the currently available suboptimal surveillance criteria [5, 24].

In conclusion, the results of the present study suggest that cardiovascular and liver-related events are important in Japanese patients with histopathologically-confirmed NAFLD. Especially, HCC was the most common liver-related event, and the incidence of HCC was more than half of that of cardiovascular events. It may be important to identify fibrosis stage and hepatocyte steatosis as determinants of HCC and T2DM, respectively. Further large-scale prospective studies should be performed to identify the predictors of development of HCC and T2DM to improve the prognosis of patients with NAFLD.

## Conclusions

Hepatocellular carcinoma was the most common liver-related event in Japanese patients with histopathologically-confirmed NAFLD.

## Abbreviations

ALT: alanine aminotransferase
AST: aspartate aminotransferase
CI: confidence interval
FLIP: Fatty Liver Inhibition of Progression
HbA1c: Glycated hemoglobin type A1c
HCC: hepatocellular carcinoma
HR: hazard ratio
NAFLD: non-alcoholic fatty liver disease
NAS: NAFLD activity score
NASH: non-alcoholic steatohepatitis
T2DM: type 2 diabetes mellitus
